# Evaluation of Pharmacological Activity of Heterobimetallic Coordination Compounds Containing* N, N*-Bis (2-hydroxyethyl)-Ethylenediamine on HT29, HeLa, C6 and Vero cells

**DOI:** 10.22037/ijpr.2019.1100854

**Published:** 2019

**Authors:** Ali Aydin, Şengül Aslan Korkmaz

**Affiliations:** a *Faculty of Art and Science, Department of Molecular Biology and Genetics, Gaziosmanpaşa University, 60240, Tokat, Turkey. *; b *Faculty of Engineering, Department of Bioengineering, Munzur University, 62000, Tunceli, Turkey.*

**Keywords:** Heterobimetallic compound, Cadmium, Cobalt, Copper, Antiproliferative, N, N-bis (2-hydroxyethyl)-ethylenediamine

## Abstract

The present study was conducted in order to investigate the pharmacological activities of three heterobimetallic coordination compounds: [Cd(*N*-bishydeten)_2_][Ni(CN)_4_] (**C1**), [Cu_2_(*N*-bishydeten)_2_Co(CN)_6_].3H_2_O (**C2**), and K[Cd(*N*-bishydeten)Co(CN)_6_].1.5H_2_O (**C3**) (*N*-bishydeten = *N,N*-bis(2-hydroxyethyl)-ethylenediamine). This paper describes the ability of complexes to inhibit cell growth, cell migration and human topoisomerase I and to interact with DNA/BSA; this paper also evaluates the potential mechanisms of action exhibited by these compounds via the use of powerful measurement techniques. Studies on HT29, HeLa, C6 and Vero cells revealed that each compound demonstrated significant antiproliferative activity in conjunction with regressed cell migration velocity and caused apoptotic changes in morphology. There are strong data suggesting that the mechanisms of action exhibited by these compounds are associated with their DNA/BSA binding features. The IC_50_ and binding constant range for the compounds are 20-180 µM and 1.2-3.2 x 10^4^ M^-1^, respectively. Moreover, we observed that these compounds alter the P53-Bcl-2 ratio and inhibit the relaxation activity of human topoisomerase I. Furthermore, a correlation between the antiproliferative effects of these compounds and their cytotoxic activity was observed. In conclusion, preliminary information demonstrates that these compounds have been found to exhibit effective antiproliferative activity against cancer cell lines, indicating that they are a potent candidate for further pharmacological study.

## Introduction

Cancer is known to exist as a heterogeneous group of various diseases that stem from different cell origins, with distinct clinical and pathological properties; these accounted for approximately 8.2 million cancer deaths across the world in 2012 alone (1). Attempting to treat cancer and to reduce its rate of occurrence is essential if we wish to lessen its burden on society. The use of various chemotherapeutic agents that kill normal cells in conjunction with cancer cells is associated with many significant problems, including the collapse of treatment, the threat of carcinogenicity, numerous unpleasant side effects such as nausea, bone marrow suppression, and kidney toxicity as well as the build-up of drug resistances, which are frequently demonstrated by patients who receive chemotherapy. This seriously hampers proper treatment, and these major drawbacks to current cancer therapies have prompted many researchers to seek alternative strategies based on various metal complexes that have not yet been extensively explored for chemotherapeutic usage. Thus, a determination of the antiproliferative features of various metal complexes with organic ligands may aid in the development of better cancer treatments and improve the quality of life for patients. Consequently, the evaluation of various metallodrug candidates that display persistent clinical effectiveness, that are linked with acceptable side effects and unassociated with drug resistance is an essential aim of research efforts. Of the metallodrug candidates, transition metal complexes such as polymer-anchored cobalt (III) complex (2) and nickel (II) complex containing thiophene based ligands (3) have been found to show activity on tumours with acquired resistance and have demonstrated unique pharmacodynamic and pharmacokinetic properties. For example, copper, which is an essential cofactor in a number of enzyme complexes, has been investigated for its DNA/BSA binding capacity and antiproliferative effects (4). Additionally, nickel is a functional constituent of some enzymes and its complexes have demonstrated a unique three-dimensional structure with organic ligands, resulting in antiproliferative activity (5). Cobalt is an essential trace element that attaches to the coring ring system of vitamin B_12_, and its associated complexes have shown considerable activity against viral, bacterial, and cancerous cells (2). Cadmium, a nonessential transition metal, is potentially toxic to humans due to its miscellaneous toxic effects. However, coordination complexes of cadmium have demonstrated potential for use in the treatment of some diseases (6, 7). These complexes have different mechanisms of action, such as the inhibition of the metastatic proteins of cancer cells (8), the facilitation of interactions with macromolecules such as DNA, RNA or protein, the progression of reactive oxygen species and the inhibition of topoisomerase I or II activity (9). Such are the significant mechanisms responsible for the anticancer effects of these complexes. Some of these complexes are used in medicines for the treatment of cancer, rheumatoid arthritis, diabetes mellitus, and parasitic diseases, and their derivatives are suitable candidates for potential alternatives to current drugs (10-13). The antiproliferative activity of transition metal complexes may stem from their DNA/BSA binding affinity and their apoptotic and anti-migratory actions; moreover, these complexes have not been fully investigated for pharmacological activity. According to this perspective, the aim of the present study was to examine the antiproliferative effects of three heterobimetallic cyanido complexes against HT29, HeLa, C6, and Vero cells and to investigate the molecular mechanisms of action exhibited by these complexes that show suppressive effects on migration and induce apoptosis. Thus, the heterobimetallic coordination compounds [Cd(*N*-bishydeten)_2_][Ni(CN)_4_] (**C1**), [Cu_2_(*N*-bishydeten)_2_Co(CN)_6_].3H_2_O (**C2**) and K[Cd(*N*-bishydeten)Co(CN)_6_].1.5H_2_O (**C3**) were successfully synthesised according to previous descriptions offered by Karadağ *et al.* (14) and Aslan Korkmaz *et al*. (15, 16). These complexes have also been studied to determine the extent of their physicochemical properties regarding DNA/BSA binding and the inhibition of restricted endonuclease activity features. The interaction of these compounds with DNA/BSA is a significant factor in the determination of the mechanisms of compound action, as DNA/BSA is a significant chemotherapeutic target for many current anticancer agents. Advanced pharmaceutical-chemical studies will provide opportunities for researchers to evaluate their pharmacological features in the treatment of diseases.

## Experimental

*Materials and Instrumentation*

NiCl_2_.6H_2_O, KCN, CuCl_2_.2H_2_O, K_3_ [Co (CN)_ 6_], CdSO_4_.8/3H_2_O and *N, N*-bis(2-hydroxyethyl)-ethylenediamine (*N*-bishydeten) were obtained commercially and used without further purification. The heterobimetallic coordination compounds [Cd(*N*-bishydeten)_2_][Ni(CN)_4_] (**C1**), [Cu_2_(*N*-bishydeten)_2_Co(CN)_6_].3H_2_O (**C2**), and K[Cd(*N*-bishydeten)Co(CN)_6_].1.5H_2_O (**C3**) (*N*-bishydeten = *N,N*-bis(2-hydroxyethyl)-ethylenediamine) were synthesized by previous method (14, 16) and elemental analyses or IR spectra used for product quality analysis and control. Elemental analyses for C, H, and N were carried out by standard methods using a CHNS-932 (LECO) Elemental Analyzer at İnönü University. IR spectra were recorded in the 4000 to 400 cm^-1^ region using a Jasco 430 FT-IR spectrometer in KBr pellets (Gaziosmanpaşa University, Turkey). Their magnetic properties, thermal analyses, and crystal structures have been previously reported (14, 16). 

*Pharmacological studies*

*Cell Culture*

Cytotoxic potential of these complexes was investigated on cancerous HT29 (Human colorectal adenocarcinoma, ATCC^®^ HTB-38™), HeLa (Human cervix adenocarcinoma, ATCC^®^ CCL-2™), and C6 cells (Rat brain glioma, ATCC^®^ CCL-107™) and nontumorigenic Vero cells (African green monkey kidney normal epithelial, ATCC^®^ CCL-81™). The cell lines were maintained in Dulbecco’s modified eagle’s medium (DMEM, Sigma) supplemented with 10% (v/v) fetal bovine serum (Sigma, Germany) and Penicillin-Streptomycin solution (10000 U / 10 mg) (Sigma, Germany) (ATCC, American Type Culture Collection). First, old medium was aspirated out of the plate while cells had reached approximately 80% confluence. Next, cells were removed from culture flask surface using 4 mL of trypsin-EDTA and centrifuged. Following, the cell pellet was resuspended with 4 mL of supplemented DMEM and was counted to gain a final concentration of 5 × 10^4^ cells/mL, and inoculated into wells (100 μL cells/well). 

*Cell proliferation inhibition assay*

A cell suspension containing approximately 5 × 10^3^ cells in 100 µL was pipetted into the wells of 96-well cell culture plates (COSTAR, Corning, USA). The cells were treated with complexes and control drug, cis-platin, dissolved in sterile DMSO (max 0.5% of DMSO) at final concentrations of 15, 30, 60, 90, 120, 150, 225 and 300 µg/mL at 37 C with 5% CO_2_ for overnight. The final volume of the wells was adjusted to 200 µL by supplemented DMEM. Cell proliferation assay was evaluated by ELISA BrdU methods as described previously (17). IC_50_ represents the concentration of an agent that is required for 50% inhibition *in-vitro*. The half maximal inhibitory concentration (IC_50_) of the test and control compounds was calculated using XLfit5 software (IDBS) and expressed in µg/mL at 95% confidence intervals. The proliferation assay results were reported as the percent inhibition of the test and control substances. The percent inhibition was calculated according to the following formula: % inhibition = [1 − (Absorbance of Treatments / Absorbance of DMSO) × 100]. The cytotoxicity of the compounds and cis-platin on HT29, HeLa, C6, and Vero cells was determined through a Lactate Dehydrogenase (LDH) Cytotoxicity Detection Kit (Roche, USA) according to the manufacturer’s instructions. Approximately, 5 × 10^3^ cells in 100 µL were seeded into 96-well microtiter plates as triplicates and treated with IC_50_ (µg/mL) concentrations of test compounds at 37 °C with 5% CO_2_ overnight. LDH activity was determined by measuring absorbance at 492 - 630 nm using a microplate reader.

*In-vitro cell death detection*

*In-vitro* detection of apoptosis was assessed on HT29 cells using a TUNEL assay kit (Roche, Germany) according to the manufacturer’s protocol. The cells (30.000 /well) were placed in a poly-L-lysine covered chamber slide and treated with the IC_50_ concentrations of test compounds at 37 ^o^C for 24 hours. The assay was carried out according to the method described by the literature (18). Briefly, cells on the chamber slide are fixed and permeabilized, and then incubated with the terminal deoxynucleotidyl transferase (TdT) and fluorescein-dUTP. TdT incorporates fluorescein-dUTP to damaged sites of the DNA. Fluorescent signal was visualized by a Leica fluorescent microscope (Leica DMIL LED fluo, Germany). 

*DNA fragmentation by agarose gel electrophoresis*

DNA fragmentation activity of the compounds was measured by using DNA laddering assay, it was described in accordance with the literature methods (19). Briefly, 7.5 x 10^5^ cells were seeded into 25 cm^2^ culture flasks, and treated with IC_50_ concentrations of test compounds at 37 °C with 5% CO_2_ for overnight. First, DNA-containing precipitate was extracted from digest with a 50 µL of phosphate-citrate buffer (consisting of 192 parts of 0.2 M Na_2_HPO_4_ and 8 parts of 0.1 M citric acid, pH 7.8), centrifuged at 1500 xg for 5 min, and then 40 µL of supernatant was transferred to a microcentrifuge tube, mixed with 5 µL of Tween20 solution (0.25% in ddH_2_O) and 5 µL of RNase A solution, and incubated at 37 °C for 30 min in a shaker incubator. Next, 5 µL of proteinase K was added to each tube and incubated at 37 °C for 10 min. Finally, DNA-containing precipitate of the micro centrifuge tube was mixed with 2 μL of 6x loading buffer, loaded to 2% agarose gel containing 0.5 μg/mL ethidium bromide and electrophoresed at 200 mA for 40 min. DNA fragmentation in the gels was visualized using gel documentation system (UVP, England). 

*Cell imaging and cell migration assay*

Cells were seeded in 96-well plates at a density of 5.000 cells per well and allowed 24 h for attachment. Using previously established IC_50_ doses of the compounds treatment was performed for 24 h and morphology changes were assessed by phase contrast microscopy. Images of vehicle (DMSO) and test compounds treated cells were taken at the end of experimental period using a digital camera attached to inverted microscope (Leica IL10, Germany). 

The migration capability of cells was measured using the migration assay as described previously (20). Briefly, an equal number of HeLa cells (3.5 x 10^4^ cells in 70 μL DMEM medium) were seeded into the two reservoirs of the same insert. Subsequent to cell growth, the insert was gently removed and 2 mL of cell culture medium was added, and then treated with 30% maximal inhibitory concentration (IC_30_) of the compounds shortly after incubated at 37 °C with 5% CO_2_. The speed of cell closure was photographed 0, 1 and 2 days after incubation using a phase contrast inverted microscope (Leica DMIL, Germany) until complete cell closure was observed in the untreated control.

*DNA topoisomerase I inhibition assay*

The DNA topoisomerase I inhibitory activities of test compounds were evaluated using a cell-free topoisomerase I assay as described previously (21). In brief, 20 µL of reaction mixture containing 0.25 µg/µL of plasmid pHOT1 DNA in relaxation buffer was incubated with 2 U recombinant human topoisomerase I enzyme in the presence of IC_50_ concentrations of the compounds, or camptothecin (CPT) as positive control. The reactions were carried out at 37 °C for 30 min and then terminated by the addition of stop solution. After the termination, the sample was analyzed using 1% agarose gel at 4 V/cm for 60 min. After electrophoresis, DNA bands were stained with ethidium bromide (EtdBr) (1 mg/mL) solution and photographed through a gel imaging system (UVP BioSpectrum, Germany).

*Immunohistochemistry*

Immunohistochemistry (IHC) techniques are used for to localize antigens changing expression level following test compounds treatment. Accordingly, HT29 and HeLa cell lines (15.000 cells/well) were placed in a poly-L-lysine covered chamber slide. The cells were treated with IC_50_ concentration of test compounds and left for 24 h of incubation. There was a negative control that had no test compound. When the incubation time was over, the chamber was removed from the slide and washed with DPBS to remove the medium and unattached cells. All of the incubation and washing steps were done in a plastic jar. The slides were gently washed with DPBS, and for fixation 4% paraformaldehyde in DPBS at pH 7.4 was freshly prepared and added to the slides for 60 min at room temperature. Following incubation, the slides were washed twice with DPBS. Heat-induced epitope retrieval (HIER) was performed using Cell Conditioning 1 (CC1), and visualization was achieved with the Universal DAB Detection Kit, according to the manufacturer’s instructions. IHC was performed using Bcl-2 (mouse monoclonal, clone 124; Ventana), CK7 (mouse monoclonal, clone OV-TL 12/30; Ventana), CK20 (mouse monoclonal, clone Ks20.8; Ventana) and P53 (mouse monoclonal, clone D07; Ventana) on the VENTANA Bench-Mark XT System. Briefly, sections were newly pretreated with CC1 Ventana reagent for 30 min at 95 °C. After pretreatment, sections were incubated with above mentioned primary antibody for 32 min at 37 °C. Reactions were revealed with an ultraView Universal DAB Detection Kit (Ventana, USA). The slides were counterstained with Hematoxylin II (Ventana) for 4 min and Bluing Reagent (Ventana) for 4 min and coverslips were applied by an automated coverslipper (Leica CV5030). For the HeLa and the HT29 cell lines, the number of positive and negative cells was counted in five zones. This procedure was repeated 3x for each protein stained slide. The slides were scored staining intensity score rated as follows: no staining (0, no stained cells or < 5% positive cells), weak staining (1+, 5–24% positive cells), moderate staining (2+, 25-49% positive cells) and strong staining (3+, > 50% positive cells). A score of 2+ or 3+ was considered positive for relevant expression while a score of 0 or 1+ was considered negative (22).

*DNA/BSA binding and gel electrophoresis studies*

UV spectroscopy was used to find the interaction of the compounds with CT-DNA and to calculate the binding constants (𝐾𝑏). A CT-DNA solution was prepared by dissolving 2.5 mg CT-DNA in 10.0 mL Tris–HCl buffer (20 mM Tris–HCl, 20 mM NaCl at pH 7.0) and stored in the refrigerator. The concentration of CT-DNA was determined spectrophotometrically using the known Ɛ value of 6600 M^−1^ cm^−1^ at 260 nm. After dissolving the CT-DNA fibers in Tris–HCl buffer, the purity of this solution was checked from the absorbance ratio A_260_/A_280_. The CT-DNA solution in the buffer displayed an A_260_/A_280 _ratio of 1.89, indicating that the DNA was sufficiently pure. These compounds were dissolved in DMSO and diluted with Tris–HCl buffer to obtain 25 µM concentrations. The test compounds in the solutions were incubated at 20 °C for about 30 min before measurements. The UV absorption titrations were conducted by keeping the concentration of these compounds fixed while varying the CT-DNA concentrations (0-100 μM). The absorption spectra were recorded by using 1-cm-path quartz cuvettes at room temperature (23). 

To evaluate the interaction of the compounds with BSA, UV spectroscopy was used. A BSA solution was prepared by dissolving 2.5 mg BSA in 10.0 mL in Tris–HCl buffer (5 mM Tris–HCl, 10 mM NaCl at pH 7.4) and stored in the refrigerator. The UV spectra of the BSA solutions (0-100 μM) in the presence of a fixed concentration of the complexes (25 μM) were scanned against the Tris–HCl buffer in the wavelength ranging from 250 to 320 nm. 

Ethidium bromide (EB) displacement experiments were performed by tracking alters in the fluorescence intensities of the EB-DNA solutions in the presence of increasing amounts of the test compound. 

The fluorescence spectra of EB were measured using an excitation wavelength of 295 nm and the emission range was set between 200 and 600 nm. The spectra were analyzed according to the Stern–Volmer equation, *I*_0_/*I* = 1 + *K*_SV_ [Q], where *I*_0_ is the fluorescence intensity in the absence of quencher, *I* is the fluorescence intensity in the presence of quencher, *K*_SV_ is the Stern–Volmer quenching constant, [Q] is the quencher concentration. *K*_SV_ can be calculated from the slope of the plot of *I*_0_/*I* vs. *[DNA]*

(24). 

The restriction enzyme inhibition assay was conducted to evaluate both specific or nonspecific binding and enzyme inhibition by the complexes. Supercoiled pTOLT (10 μM) plasmid DNA was incubated with the complexes (25 μM) and restriction enzymes *Kpn*I and *Bam*HI (2 units) at 37 °C in 50 mM Tris–HCl/18 mM NaCl buffer (pH 7.2) for 4 h. The digestion products were resolved by using 1.5% (wt/vol) agarose gels with ethidium bromide. 

*Statistical analysis*

The statistical significance of differences was determined by one-way analysis of variance (one-way ANOVA) tests. Post hoc analyses of group differences were performed using the Tukey test, and the levels of probability were noted. SPSS for Windows was used for statistical analyses. The results are reported as the mean values ± SEM of three independent assays, and the differences between groups were considered to be significant at *P *< 0.05.

## Results

*IR Spectra*

The tentative assignments and the wavenumbers of the vibrational modes of functional groups in the complexes are presented in the experimental section. The IR spectra exhibiting sharp bands at 2128 cm^-1^ for **C1** and 2156 cm^-1^ for **C3** are attributed to the terminal cyanido groups, while these absorption bands at 2128 cm^-1^ and 2129 cm^-1^ are for free [Ni(CN)_4_]^2-^ and [Co(CN)_6_]^3-^, respectively. In the spectrum of **C2** three peaks are observed at 2173, 2154, 2133 cm^-1^ and this situation is in agreement with existence of the crystallographically different cyanido groups in its structure. These wavenumbers assigned to the **C1**, **C2** and **C3** are in good agreement with the values reported in the literature (14-16).

*X-ray study*

The crystal structure is ionic and consists of [Cd (C_6_H_16_N_2_O_2_)_2_]^2+^ cations and [Ni (CN)_4_]^2-^ anions as described in the literature (14). Aslan Korkmaz *et al*. (16) reported that the asymmetric unit of polymeric **C2** consists of one trinuclear-CN-Cu1 (C_6_H_16_N_2_O_2_)-µ-O-Cu2 (C_6_H_16_N_2_O_2_)-NC-Co(CN)_4_-CN-{Cu1Cu2Co3} unit and three water molecules in a one dimensional zigzag chain. The most important feature of **C2** structure is the connection with *µ*^1^-O2 between the Cu1 and Cu2 atoms.

*Inhibition of cell proliferation and cytotoxicity*

**Figure 1:**
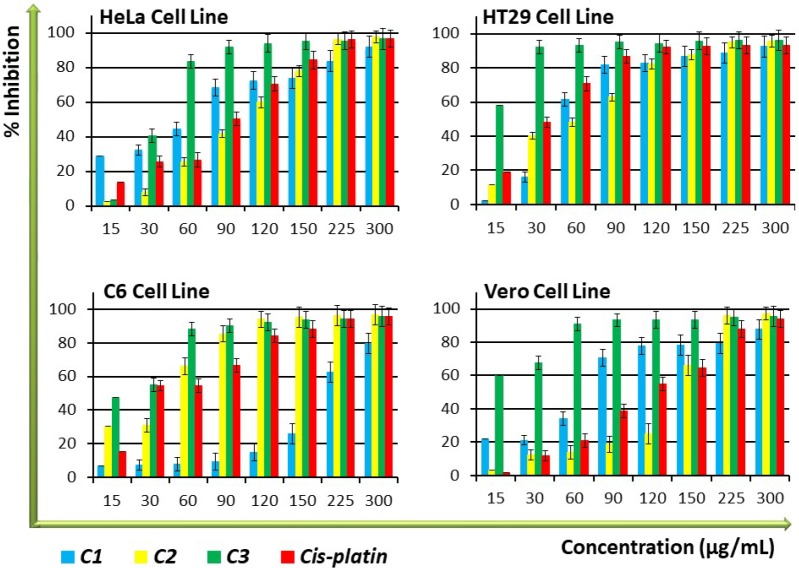
Antiproliferative activity of **C1-C3 **and positive control compound, cis-platin on HT29, HeLa, C6, and Vero cell lines. Percent inhibition was reported as mean values ± SEM of three independent assays (*P *< 0.05). Each experiment was repeated at least three times for each cell line

The amount of DNA in each cell is kept constant, thus DNA quantity can provide relevant information on cell numbers. To determine whether the compounds have shown any effect on the proliferation of cells, a BrdU Cell Proliferation ELISA measurement of the amount of DNA was performed on the cell lines. As shown in Figure 1, **C3** demonstrated significantly (*P* < 0.05) higher antiproliferative activity than cisplatin, the control anticancer drug, against the tested cell lines, especially at high concentrations made in a dose-dependent manner. While **C1** displayed higher antiproliferative activity than cisplatin against HeLa and Vero cell lines, **C2** exhibited antiproliferative activity that was equal to cisplatin, especially in the low and medium concentration groups. Antiproliferative activity of **C1 **and **C2** was lower on nontumourigenic Vero cells compared to cancer cells (Figure 1 and Table 1), indicating an interesting selectivity towards the cancer cells. The IC_50_ values of the compounds to be used in consequent tests were determined by performing ELISA BrdU assay data calculations, as displayed in Table 1. The data on cell proliferation inhibition demonstrated that the antiproliferative effects of C1, C2, and cisplatin are nearly identical, but that their selectivity is lower than that of cisplatin (Figure 1 and Table 1).

**Figure 2:**
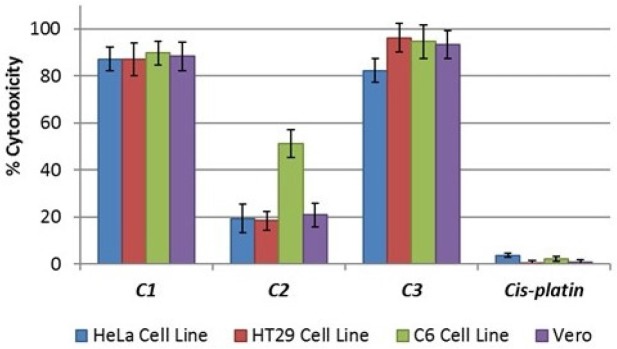
Cytotoxic activity of **C1-C3 **on HT29, HeLa, C6, and Vero cell lines. Percent cytotoxicity was reported as mean values ± SDs of three independent assays (*P *< 0.05)

Lactate dehydrogenase (LDH) is a stable cytoplasmic enzyme that is normally found in all cells. Cytotoxicity can be determined by measuring the LDH that is released into the supernatant from damaged plasma membranes. Therefore, the cytotoxic activity of the compounds on HT29, HeLa, C6, and Vero cell lines was tested using an LDH cytotoxicity assay kit and was determined via the overnight treatment of the cells with IC_50_ concentrations of the various compounds; these tests revealed the concentration-dependent detriment of the cell membranes. The data on cytotoxicity percentages indicated that the treatment of cells with IC_50_ concentrations of **C1** and **C3** resulted in the significant corruption of cell membrane integrity as compared to cisplatin (Figure 2). **C2** had no cytotoxicity in the cells at IC_50_ concentrations, but exhibited potent cytotoxic activity towards the C6 cell line (Figure 2).

*Apoptotic potential*

**Figure 3:**
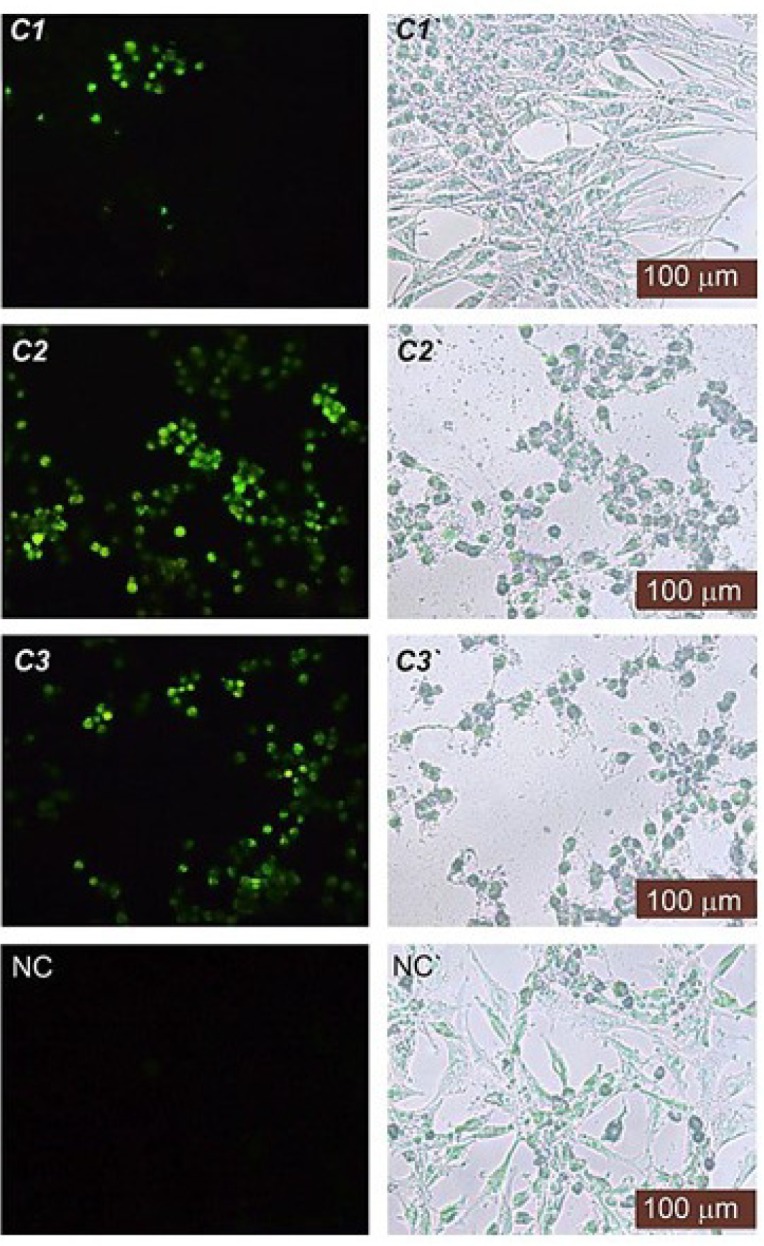
Fluorescence (left side) and phase-contrast (right side) images of the HT29 cancer cells treated with **C1-C3 **at IC50 concentration, DMSO (NC) after TUNEL assay. TUNEL-positive cell nuclei were observed in brilliant green under fluorescence. NC (negative control)

Terminal deoxynucleotidyl Transferase (TdT) is a type of DNA polymerase that is found in immature lymphoid cells. This enzyme (TUNEL reaction) preferentially uses the fluorescence-dUTP to label DNA strand breaks that occur during apoptosis. We performed a TUNEL assay to ascertain whether the test compound-induced inhibition of cell proliferation was associated with cell apoptosis. The TUNEL reaction would prefer to mark DNA strand breaks generated during apoptosis as compared to necrosis. As illustrated in Figure 3, the compounds, especially the **C2** and **C3** treated cells, displayed a higher percentage of TUNEL-positive apoptotic cell nuclei (*P* < 0.05)—thus indicating the presence of apoptotic DNA—whereas the DMSO control contained TUNEL-negative cell nuclei. For each concentration, the apoptotic index was determined by counting the percentage of TUNEL-positive cells from at least 100 nuclei. The apoptotic index was roughly > 30% for **C2** and **C3** at IC_50_ concentration and approximately > 50% for **C1** at IC_50_ concentration.

*DNA fragmentation*

**Figure 4:**
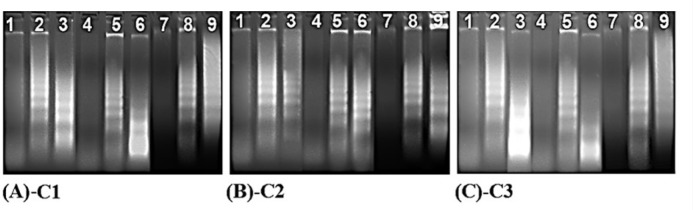
The effects of **C1 **(A), **C2 **(B) and **C3 **(C) on DNA fragmentation in non-treated control (1, 4, 7), positive control treated with camptothecin (2, 5, 8) and sample treated with the compounds (3, 6, 9) of HeLa, HT29 and C6 cancer cell lines, respectively

DNA fragmentation is a significant marker of cellular apoptosis. In this process, genomic DNA was cleaved onto the nucleosome oligomers of about 180–200 base pairs via a caspase-activated DNase (CAD). These oligomers appeared as a DNA ladder on an agarose gel. The cells were subjected to IC_50_ concentrations of the compounds to further confirm the cell apoptosis revealed by these compounds. As shown in Figure 4, DNA laddering patterns were photographed in the cells treated with IC_50_ doses of the compounds. Hence, all compounds significantly induced the formation of DNA fragmentations (a sign of apoptosis) in HT29, HeLa, C6, and Vero cell lines as compared to the control cells (Figure 4). 

*Cell morphology*

The morphology of test compound-treated cells revealed typical features of apoptosis such as cell shrinkage, the formation of apoptotic bodies, chromatin condensation, and the atypical shape of floating cells; these are all hallmarks of the cell death. The compounds significantly inhibited the growth of the cells in the cultures. The control groups, however, showed complete confluent growth and normally proliferating cells.

**Figure 5:**
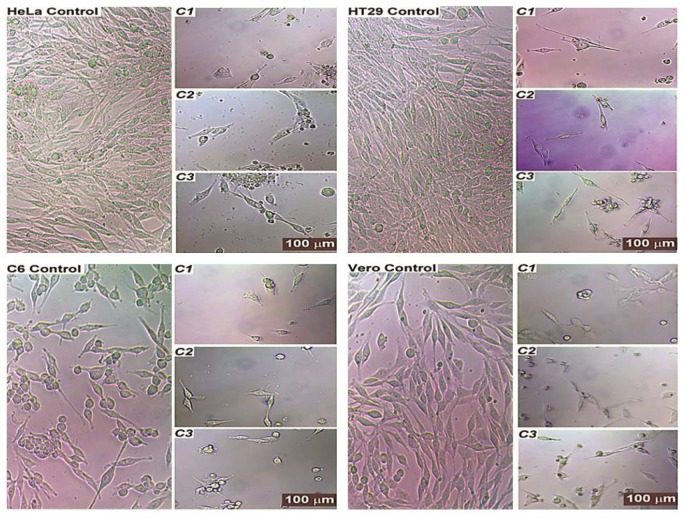
The effects of **C1-C3 **on the morphology of HT29, HeLa, C6, and Vero cells. Exponentially growing cells were incubated with IC50 concentrations of the compounds at 37 °C for overnight. DMSO treated cells as controls

Most of the treated cells had an abnormal fibroblast-like appearance and were detached from the plate; while this resolved upon treatment, the number of cells seemed to be lower and the cells began to separate from one another and to appear smaller. Treatment with test compounds also showed the significant detriment of cells, as cell growth was affected and cellular morphology became similar to that seen in apoptotic situations. The number of cells also decreased and cells began to appear structurally different from untreated cells. The results in Figure 5 demonstrate the normal structure of the most control cells that effectively grew in the medium.

*Cell migration*

**Figure 6:**
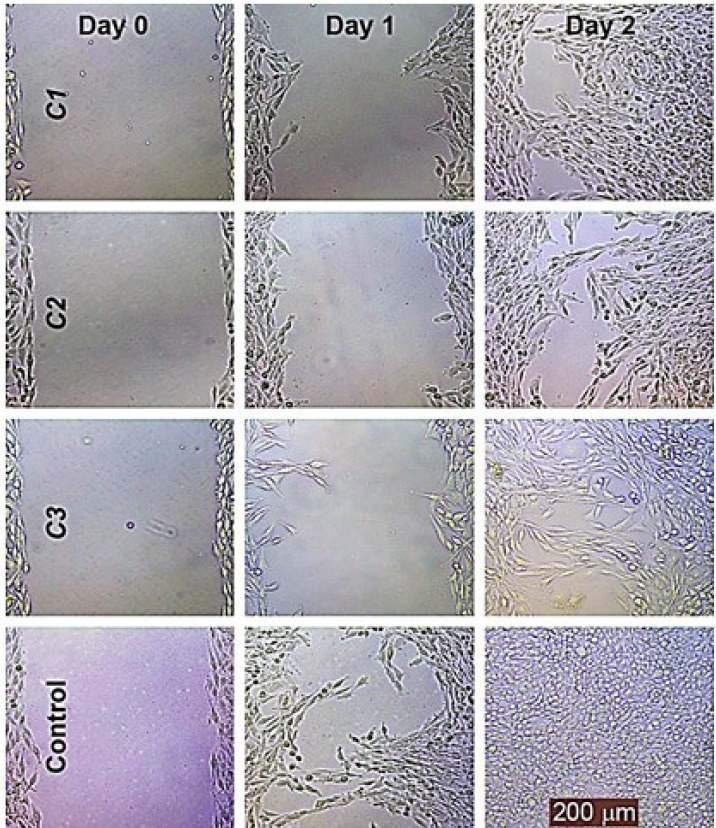
The effects of **C1-C3 **on the migration of the HeLa cell line. The wound healing of the HeLa cell line was photographed at 0, 1 and 2 days following incubation with these compounds at 30 % maximal inhibitory concentration (IC30) using a phase contrast microscope

The representative photographs depicted in Figure 6 show the lower migration capability of HeLa cells treated with these compounds at 30% maximal inhibitory concentration (IC_30_) as compared to the untreated controls using the migration assay. 

These compounds dramatically inhibited HeLa migration in a concentrated and time-dependent manner; it should be noted that the lowest effective concentration was 20% maximal inhibitory concentration (IC_20_) in the migration assay.

*DNA topoisomerase I inhibitory activity of C1, C2 and C3*

DNA topoisomerase I is a nuclear enzyme that plays an important role in the control of topological state DNA; this enzyme is essential to cell viability. Therefore, DNA topoisomerase I is an important target of current medicinal agents such as camptosar, irinotecan, and topotecan. Thus, in order to better understand whether the antiproliferative activity of these compounds involves the inhibition of DNA topoisomerases, we investigated the effects of our compounds on the recombinant human topoisomerase I-mediated relaxation of supercoiled plasmid (pHOT1) DNA.

**Figure 7:**
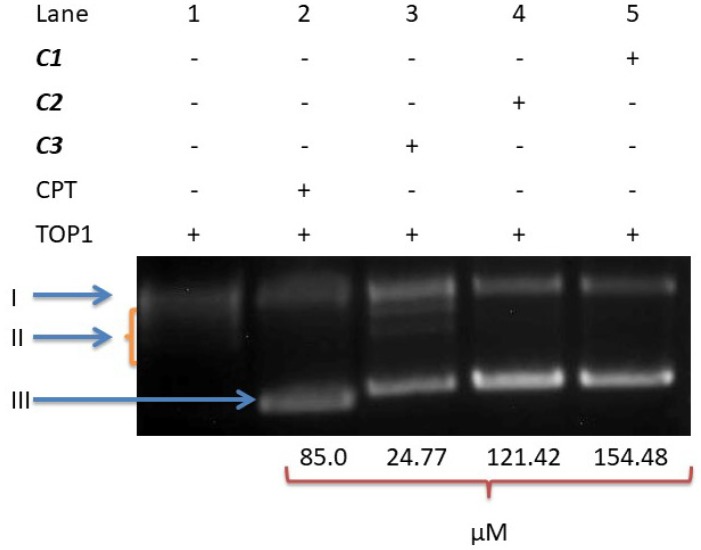
The DNA unwinding analysis with **C1-C3**. A DNA unwinding assay was performed with 250 ng pHOT-1 supercoiled DNA, 2U TOP1 and IC50 concentrations of the compounds. The forms of DNA are denoted as I (Nicked DNA), II (Relaxed DNA), and III (Supercoiled DNA)

As shown in Figure 7, the IC_50_ concentrations of **C1** and **C2** used in this experiment inhibited the DNA relaxation activity of DNA topoisomerase I; **C3**, however, did not inhibit this enzyme activity. 

*IHC evaluation of slides treated by the complexes*

**Figure 8:**
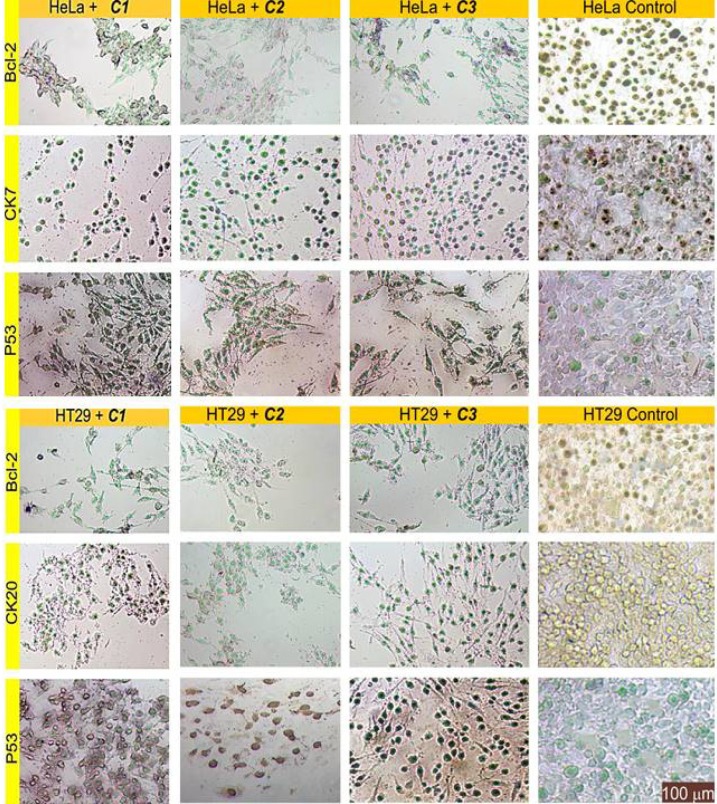
Representative images of the cells examined by immunohistochemical staining for functional protein group (Bcl-2 and P53), and for marker protein group (CK7 and CK20). The specific signals are shown as brown staining

Immunohistochemistry staining of the sectioned slides showed the decreased expression of Bcl-2 and the increased expression of P53 as an expected observation in points of cell survival in complex-treated cells; this emphasises the deadly effects of these compounds (Figure 8). There are closed aspects of these findings in relevant literature (6, 13).

The results also depicted that delivery of the complexes into cells caused a significant reduction in the expression of CK20 and CK7. This situation can be associated with reduced metastatic ability due to the anti-migratory effects of these compounds.

*DNA/BSA binding and gel electrophoresis studies*

**Figure 9:**
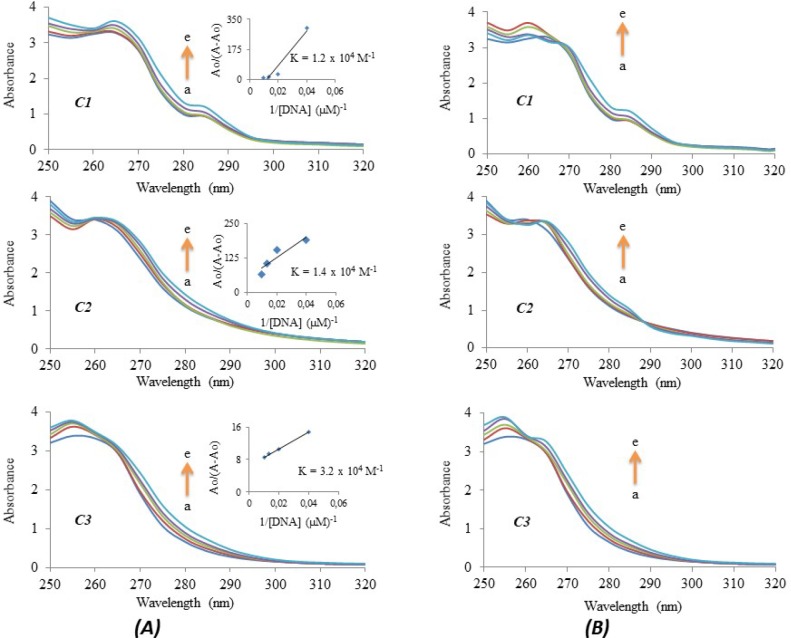
(***A***) UV–Visible absorption spectra of 25 µM **C1**, **C2 **and **C3 **in the absence (a) and presence of 25 μM (b), 50 μM (c), 75 μM (d) and 100 μM (e) DNA. Note: The direction of arrow demonstrates increasing concentrations of DNA. Inside graph is the plot of *A0/ (A–A0) *vs. *1/[DNA] *to find the binding constant of the complex–DNA adduct. (***B***) Absorption spectra of 25 µM **C1**, **C2 **and **C3**

Complex-DNA interactions can be investigated via a comparison of UV-V absorption spectra of the free complex and complex-DNA adducts (25). The binding constant (*K*) of **C1**, **C2** or **C3 **with DNA can be determined according to the Benesi-Hildebrand equation,*A*_0_*/A-A*_0_*=Ɛ*_G_*/Ɛ*_H–G_*-Ɛ*_G_*+Ɛ*_G_*/Ɛ*_H–G_*-Ɛ*_G_*x1/K[DNA]*, where K is the binding constant, *A*_0_ and *A* represent the absorbances of either **C1**,** C2**, or** C3 **and its adduct with DNA and *Ɛ*_G_ and also *Ɛ*_H–G_ signify the absorption coefficients of the complexes and the complex-DNA adducts (25). The binding constant can be obtained from the intercept-to-slope ratio of *A*_0_*/ (A - A*_0_*)* vs. *1/[DNA]* plots. Figure 9A describes the interaction of these complexes with CT-DNA. According to the Benesi-Hildebrand equation, the plot of *A*_0_*/(A–A*_0_*)* vs. *1/[DNA]* data yielded the binding constant (*K*), which was 1.2 x 10^4^ M^-1^ for ***C1***, 1.4 x 10^4^ M^-1^ for ***C2*** and 3.2 x 10^4^ M^-1^ for ***C3*** (Figure 9A). The increase in CT-DNA concentrations resulted in hyperchromic effects in the absorption bands, with a moderate red shift indicating a strong interaction between the complexes and DNA. This hyperchromic effect on the spectra of the complex-DNA adduct might be indicative of groove binding.

An understanding of the properties associated with the complex interactions of bovine serum albumin (BSA), which is the main carrier protein in the circulatory system, can provide more information about pharmacological features. The interaction of these complexes with BSA can be observed via a comparison of UV-Visible absorption spectra and the emission spectra of the free complex and complex-BSA adducts. The absorption spectra of the BSA solutions (0-100 μM) in the absence and presence of the complexes (25 μM) are shown in Figure 9B. The complexes caused an increase in the absorbance of BSA and exhibited a slightly blue shift, indicating Van der Waals contacts or hydrogen bonds during interaction with BSA. Isosbestic points near 265 nm and 290 nm for **C2** and 268 nm for **C1** were observed.

**Figure 10:**
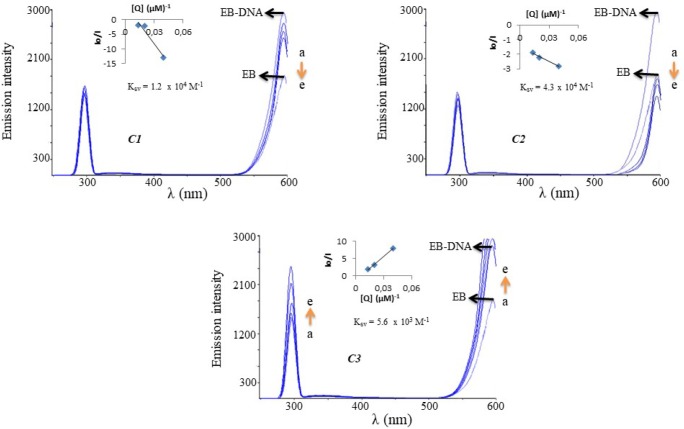
The emission spectra of EB-bound (a) DNA solutions in the absence and presence of increasing concentrations of **C1-C3 **25 μM (b), 50 μM (c) and 75 μM (d). [EB]=10.0 μM (a), [DNA] 50.0 μM. The arrows show the changes in intensity upon increasing amounts of **C1**, **C2 **or **C3**. Insets: Stern–Volmer plot of the fluorescence data. Inset shows the plots of emission intensity *I0/I *vs. *[Q] *(µM) for determining *KSV*

The emission spectra of EB bound to DNA in the absence and presence of the complexes is shown in Figure 10, where the fluorescent intensity of EB-DNA decreased in the presence of a second molecule that intercalated itself onto the base pair of the DNA. The fluorescent quenching of EB bound to CT-DNA by these complexes is shown in Figure 10.

The quenching parameters of EB bound to CT-DNA by these complexes were evaluated using the linear Stern-Volmer equation (*I*_0_/*I* = 1 + *K*_SV_ [Q]), which provided further evidence that the complexes bind to DNA (25). The *K*_SV_ values for **C1**, **C2** and **C3** are 1.2 x 10^4^ M^-1^, 4.3 x 10^4^ M^-1^, and 5.6 x 10^3^ M^-1^, respectively. The data suggested that the interaction of **C3** with CT-DNA was the strongest of these three complexes. Interestingly, the **C3** complex caused obvious increases in emission intensity, indicating that the **C3** complex replaced EB when binding to CT-DNA, thus acquiring fluorescent features.

**Figure 11:**
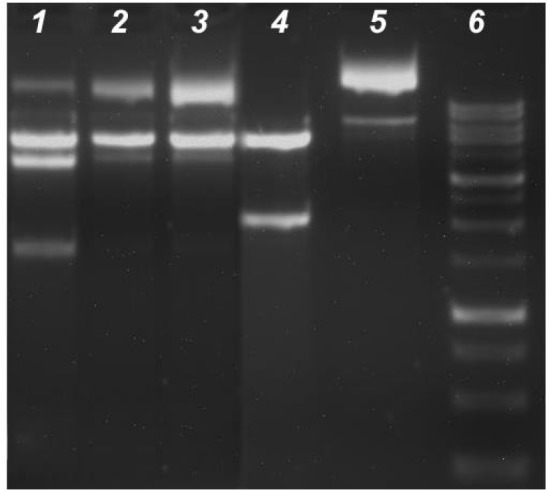
The inhibition of *Kpn*I and *Bam*HI restriction endonucleases activity. Following 4 h 37°C digestion of the 14µL with 10 U*Kpn*I and *Bam*HI, these digestion products were resolved with 1.5% agarose gel containing ethidium bromide. Lane 1: enzyme + DNA+ **C3**, Lane 2: enzyme + DNA + **C2**, Lane 3: enzyme + DNA + **C1**, Lane 4: Positive control (enzyme + DNA), Lane 5: Negative control (plasmid DNA + water); lane 6: DNA marker (1Kb)

After *Kpn*I and *Bam*HI digestion of pTOLT plasmid DNA, the digestion products were identified by two DNA bands in the absence complexes (Lane 4), whereas four bands (Lane 1) were produced in the presence of **C3** and three bands were displayed in the DNA fragments of** C1** and **C2** (Lane 2 and Lane 3) (Figure 11). In the presence of **C3**, DNA digestion was incomplete and the new bands were observed near the well at the top of the lane (Lane 1).

Treatment of *Kpn*I and *Bam*HI with **C1 **or **C2 **strongly inhibited the restriction of endonuclease activity in these enzymes; thus, three bands corresponding to the supercoiled and nicked DNA were observed in the undigested DNA (Lane 5). The results indicated that **C3** likely bound to pTOLT plasmid DNA. However, **C1 **and** C2** most likely bound to these enzymes, which resulted in the strong inhibition of restricted endonuclease activity in* Kpn*I and *Bam*HI (Lane 2 and Lane 3).

(a) in presence of different concentrations of BSA 25 μM (b), 50 μM (c), 75 μM (d), and 100 μM (e). Note: The direction of arrow demonstrates increasing concentrations of BSA.

**Table 1:** IC50 values and tumor specificity rate for **C1**, **C2**, **C3**, and Cis-platin

**Compounds**	**IC** **50 ** **(µM)**			
	** HeLa**	** HT29 **	** C6**	** Vero**
**C1**	159.27	154.48	364.58	177.92
**C2**	176.72	121.42	86.67	210.47
**C3**	100.41	24.77	49.47	22.17
**Cis-platin**	352.12	167.66	233.89	447.48

## Discussion

In this study, we have evaluated the pharmacological activity of [Cd(*N*-bishydeten)_2_][Ni(CN)_4_] (**C1**), [Cu_2_(*N*-bishydeten)_2_Co(CN)_6_].3H_2_O (**C2**) and K[Cd(*N*-bishydeten)Co(CN)_6_].1.5 H_2_O (**C3**) (*N*-bishydeten=*N,N*-bis(2-hydroxyethyl)-ethylenediamine) and outlined the possible instruments responsible for their mechanisms of action using different molecular techniques. These findings prove that these compounds can inhibit cell proliferation at 30 % maximal inhibitory concentration (IC_30_), induce apoptosis through DNA fragmentation and increase the inhibition of migration, thus creating new opportunities for cancer investigation. Cell proliferation assay results showed that **C3** was significantly more antiproliferative against all cell lines than the control drug cisplatin (Figure 1), indicating its antitumour potential. The antiproliferative effect of **C1** was higher on HeLa and Vero cells than on HT29 and C6 cells and was also statistically significant (*P* < 0.05). **C2** had an antiproliferative activity that was equal to cisplatin, especially at low and medium concentrations. Similar to other reports using various methods (26, 27), all compounds generally exhibited significant antiproliferative effects. 

Cytotoxic effects led to undesirable increases in the LDH leakage of plasma membranes. Hence, LDH assay results implied that **C1** and **C3** showed cytotoxic effects on the cells, but that **C2** caused a cytostatic effect on the cell lines (Figure 2). From this perspective, our findings indicated that **C2** exerts high antiproliferative and low cytotoxic activities towards cell lines; this suggests that **C2** has significant potential as a useful medicinal agent. As emphasised in several previous studies, these compounds have effects similar to those of current chemotherapeutic agents, as reported arious cell lines, including A549 cells and CDDP-resistant gastric cancer cell lines (28, 29). Moreover, these studies have also showed that these compounds exhibit pharmacological effects via apoptotic pathways rather than other possible mechanisms.

Apoptosis induction via these compounds is confirmed by DNA laddering and TUNEL assays. First, the DNA laddering assay depicts DNA laddering patterns that are considered to contain apoptotic characteristics and to be supplied by endonuclease activity. Chemotherapeutic agents are typically considered to affect tumour cells by activating apoptotic pathways, resulting in cell death. Consistent with this line of thought, we have provided evidence that cells treated with these compounds at IC_50_ exhibit a ladder pattern characteristic of apoptosis (Figure 4). DNA fragments that appear during apoptosis have been previously shown to occur in other coordination compound-treated cells, such as in SKW-3 (T-cell leukaemia), K-562, LAMA-84 (chronic myeloid leukaemia), and 5637 (urinary bladder cancer) (30). Second, a TUNEL assay was used to determine whether apoptosis was involved in the mechanism of action of these compounds in HT29 cells. The number of the TUNEL-positive cells suggested the existence of DNA fragmentation in the cells. As depicted in Figure 3, the treated cells were TUNEL-positive, indicating that these compounds significantly induced apoptosis in HT29 cells. A similar observation was made in previous reports, which revealed that the coordination compounds induced apoptosis via the activation of caspase cascades, thus causing DNA fragmentation (31). Therefore, our TUNEL assay results were also consistent with our DNA laddering assay results, indicating that these compounds caused DNA fragmentation (Figure 4). Overall, these results demonstrated that these compounds could exhibit a robust apoptotic effect on HT29 cells.

These compounds decreased cell growth in cultures by causing cell shrinkage, leading cells to become smaller, swell, and float with an abnormal shape (Figure 5). The treated cells transformed, shedding their astrocyte-like and fibroblast-like structures and taking on a globular shape that was detached from the plate. The number of the cells also reduced, and treated cells looked structurally different from untreated cells; treated cells began to separate from one another and appear smaller. 

The migration capacity is considered an important target for chemotherapy, as the migrating cancer cells are resistant to apoptosis. Several chemotherapeutic agents approve the inhibition of cell migration. Hence, an antiproliferative agent with antimigratory activity may be preferable for cancer treatment. These compounds, at 30 % maximal inhibitory concentration (IC_30_), are able to inhibit the migration ability of HeLa cells (Figure 6). However, there was a major difference in the migration rate of these compound treatments at concentrations lower than 20 % maximal inhibitory concentration (IC_20_) and higher than 50 % maximal inhibitory concentration (IC_50_) when compared with the control cell line (data not shown). According to the results, treatment with these compounds inhibited the migration of HeLa cells by decreasing the cell growth rate in a concentrated and time-dependent manner. Similar metal compounds have also been reported to strongly restrict cell migration, resulting in modulated cell death or apoptotic stress (32-34). 

According to the relevant literature information, antitopoisomerase activity depends primarily on the metal-DNA or metal-topoisomerase interaction type and on the physicochemical features of the metal compounds (solvent polarity or ionic strength) (35, 36). The effect of these complexes on human DNA topoisomerase I showed that **C1** and **C2** inhibited the DNA relaxation activity of DNA topoisomerase I, but that **C3** failed to affect this relaxation activity. The results may indicate that **C1** and **C2** regressed cell proliferation via the suppression of DNA topoisomerase I action or via the provocation of metal-DNA damage during the replication, but that **C3** blocks cell proliferation via a process other than enzyme inhibition (Figure 7). This finding may also suggest the binding of these complexes not only to topoisomerase I but also to other proteins such as BSA, Bcl-2, and P53 (Figure 7, Figure 8 and Figure 9). In fact, the administration of **C1**–**C3** to cells resulted in a loss of Bcl-2 and an accumulation of P53. This change of Bcl-2 and P53 levels resulted in the trigger of apoptosis, thus bringing about the therapeutic action of **C1**–**C3**. 

Because metal complex-DNA/BSA interactions have proved to be powerful acts, this study also researched possible structural forms of the metal complex-DNA/BSA. The binding of the complexes to CT-DNA and to BSA resulted in significant changes to spectral characteristics. The complexes showed hyperchromic absorption spectra and a slight red shift that interacted directly with CT-DNA through a groove or intercalative binding mode. The spectral data suggest a force that acts between the complexes and indicates that BSA is mainly a hydrophobic, Van der Waals contact, or hydrogen bond interaction. Thus, the complexes interact effectively with BSA. It is clear that these physiochemical interactions of metal complexes can reveal their binding affinity and selectivity (37). Moreover, the complexes caused the partial inhibition of restricted endonuclease activity in* Kpn*I and *Bam*HI, which indicated that the complexes can interact with CT-DNA, with these enzymes or with both factors. To sum, we have evaluated that the strong apoptosis inducers **C1**–**C3** possess vigorous inhibitory effects on the cancer cell lines *in-vitro*. Based on our results, it is suggested that **C1**–**C3** are potentially valuable cytotoxic agent candidates and are suitable for further pharmacological testing. 
